# Melatonin-Induced Protection Against Plant Abiotic Stress: Mechanisms and Prospects

**DOI:** 10.3389/fpls.2022.902694

**Published:** 2022-06-09

**Authors:** Muhammad Umair Hassan, Athar Mahmood, Masood Iqbal Awan, Rizwan Maqbool, Muhammad Aamer, Haifa A. S. Alhaithloul, Guoqin Huang, Milan Skalicky, Marian Brestic, Saurabh Pandey, Ayman El Sabagh, Sameer H. Qari

**Affiliations:** ^1^Research Center on Ecological Sciences, Jiangxi Agricultural University, Nanchang, China; ^2^Department of Agronomy, University of Agriculture Faisalabad, Faisalabad, Pakistan; ^3^Department of Agronomy, Sub-Campus Depalpur, Okara, University of Agriculture Faisalabad, Faisalabad, Pakistan; ^4^Biology Department, Collage of Science, Jouf University, Sakaka, Saudi Arabia; ^5^Department of Botany and Plant Physiology, Faculty of Agrobiology, Food, and Natural Resources, Czech University of Life Sciences Prague, Prague, Czechia; ^6^Department of Plant Physiology, Slovak University of Agriculture, Nitra, Slovakia; ^7^Department of Agriculture, Guru Nanak Dev University, Amritsar, India; ^8^Department of Agronomy, Faculty of Agriculture, Kafrelsheikh University, Kafr El-Sheikh, Egypt; ^9^Department of Field Crops, Faculty of Agriculture, Siirt University, Siirt, Turkey; ^10^Department of Biology, Al-Jumum University College, Umm Al-Qura University, Makkah, Saudi Arabia

**Keywords:** abiotic stress, anti-oxidant defence, growth, genes regulation, melatonin, ROS, signalling crosstalk

## Abstract

Global warming in this century increases incidences of various abiotic stresses restricting plant growth and productivity and posing a severe threat to global food production and security. The plant produces different osmolytes and hormones to combat the harmful effects of these abiotic stresses. Melatonin (MT) is a plant hormone that possesses excellent properties to improve plant performance under different abiotic stresses. It is associated with improved physiological and molecular processes linked with seed germination, growth and development, photosynthesis, carbon fixation, and plant defence against other abiotic stresses. In parallel, MT also increased the accumulation of multiple osmolytes, sugars and endogenous hormones (auxin, gibberellic acid, and cytokinins) to mediate resistance to stress. Stress condition in plants often produces reactive oxygen species. MT has excellent antioxidant properties and substantially scavenges reactive oxygen species by increasing the activity of enzymatic and non-enzymatic antioxidants under stress conditions. Moreover, the upregulation of stress-responsive and antioxidant enzyme genes makes it an excellent stress-inducing molecule. However, MT produced in plants is not sufficient to induce stress tolerance. Therefore, the development of transgenic plants with improved MT biosynthesis could be a promising approach to enhancing stress tolerance. This review, therefore, focuses on the possible role of MT in the induction of various abiotic stresses in plants. We further discussed MT biosynthesis and the critical role of MT as a potential antioxidant for improving abiotic stress tolerance. In addition, we also addressed MT biosynthesis and shed light on future research directions. Therefore, this review would help readers learn more about MT in a changing environment and provide new suggestions on how this knowledge could be used to develop stress tolerance.

## Introduction

Plants are sessile organisms that face a variety of environmental stress (drought, salinity, heat, cold stress, heavy metals stress, and nutrient deficiency) (Rasheed et al., 2021a,b), which have devastating impacts on their performance in terms of growth and productivity (Sharma et al., 2019). These abiotic stresses disrupt plant physiological and metabolic functioning development processes (Jeandroz and Lamotte, 2017) and induce the production of reactive oxygen species (ROS), lipid peroxidation and accumulation of various osmolytes, and significant yield losses (Arif et al., 2016; Singh et al., 2017; Batool et al., 2022a,b; Imran et al., 2022). The intensity of these abiotic stresses is steadily increasing due to rapid climate change, and appropriate measures need to be taken to address these stresses (Beebe et al., 2011; Jeandroz and Lamotte, 2017; Ali et al., 2019). Therefore, plants have developed diverse mechanisms to counter these abiotic stresses (Rasheed et al., 2020a,b). Such tools include plant growth regulators, different osmolytes synthesis, and accumulation to protect against stress-induced damages for maintaining cellular homoeostasis and optimum plant growth (Yancey, 2005; Burg and Ferraris, 2008; Beebe et al., 2011; Liang et al., 2013; Singh et al., 2017).

Melatonin (MT) is one such molecule considered a vital plant growth regulator under stress conditions. It is a pineal molecule discovered in bovine pineal glands (Lerner et al., 1958; Reiter, 1991). MT received its name in 1957 when it was reported to play a role in the skin lightening of frogs and involves in controlling circadian rhythms in diverse vertebrates (Lerner et al., 1958; Tan et al., 2018). The maximum MT levels during the night indicate its importance in nocturnal signalling (Reiter, 1991). In plants, the MT presence was discovered in various monocot and dicot families (Reiter et al., 2001; Nawaz et al., 2016). Its presence in diverse plant parts (root, stem, leaves, fruit, flower, and seeds) in apple, banana, cucumber, onion, rice, and tomato, indicates its importance in plant growth and development across the plant kingdom (Nawaz et al., 2016; Wei et al., 2018).

The MT role in response to different stresses has been comprehensively studied (Debnath et al., 2019). MT plays an important role in seed germination, biomass productivity, photosynthesis, fruit maturation, membrane integrity, osmoregulation, leaf senescence and plants responses to abiotic stresses (Lee et al., 2014; Shi et al., 2015b). MT-mediated gene expression regulation protects plants against stress conditions, for example, the activation of antioxidant machinery of plants (Debnath et al., 2019); thus, it is considered an essential bio-stimulant to improve crop production in adverse conditions. MT triggered an antioxidant defence system under stress conditions, favouring ROS scavenging and acting as a stress protecting molecule (Khan et al., 2020a). This property of MT makes it a promising molecule that can be used for exogenous application under stress conditions. In this review, we have explored the physiological and biochemical role of MT under diverse abiotic stresses. We also discussed the possible mechanism of MT under different stresses. Moreover, we have also shed light on engineered MT biosynthesis, its crosstalk with other hormones, and future research to provide a complete picture of MT-mediated abiotic stress tolerance.

## Biosynthesis of Melatonin in Plants

**FIGURE 1:**
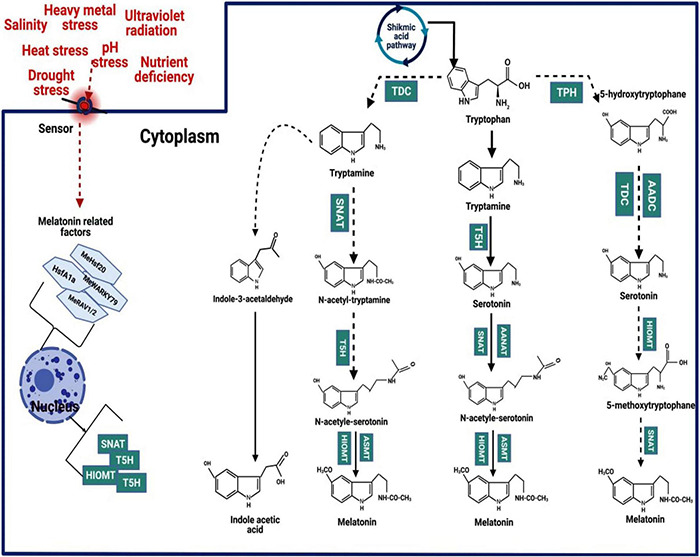
Mechanism of melatonin biosynthesis in the plant.

In the MT biosynthesis pathway, the tryptophan (TTP) precursor, which is also a precursor of indole-3-acetic acid (IAA), comes from the shikimic acid pathway (Posmyk and Janas, 2009; Arnao and Hernández-Ruiz, 2014; Nawaz et al., 2016; Zhao et al., 2019). The TTP is converted in MT by four enzymatic reactions catalysed by four diverse enzymes (Figure 1). The enzyme tryptophan decarboxylase (TDC) firstly changed TTP into tryptamine. After that, the enzyme tryptamine 5-hydroxylase (T5H) converts tryptamine into serotonin. These two steps are crucial for the synthesis of serotonin in plants. Nevertheless, in some plants, a different pathway operates in which tryptophan is converted by tryptophan 5-hydroxylase (TPH) to 5-hydroxytryptophan, which is then catalysed by tryptophan decarboxylase or aromatic L-amino acid decarboxylase (TDC/AADC) to serotonin (Zuo et al., 2014). Subsequently, arylalkylamine N-acetyltransferase (AANAT) or N-acetyltransferase (SNAT) converts serotonin into N-acetyl-serotonin. Moreover, SNAT can also convert tryptamine into N-acetyl-tryptamine; however, T5H cannot convert N-acetyl-tryptamine into N-acetyl-serotonin. In the last step, N-acetyl-serotonin methyltransferase (ASMT) or hydroxyindole-O-methyltransferase (HIOMT) catalysed the N-acetyl-serotonin into MT. HIOMT can also convert serotonin into 5-methoxytryptamine, converted into MT by SNAT (Zhang et al., 2014; Tan et al., 2016).

Generally, MT and its intermediate accretion in different sub-cellular sites depend on the order of enzymes reaction involved in MT biosynthesis. For instance, the accumulation of serotonin occurs in the endoplasmic reticulum when TTP is converted into T5H, while serotonin accumulates in the cytoplasm in the TDC enzyme. Likewise, the conversation of serotonin into N-acetyl-serotonin occurs in the chloroplast, where serotonin conversion into 5-methoxytryptamine by ASMT accumulation occurs in the cytoplasm. Finally, MT synthesis occurs in the chloroplast (Miller et al., 2010). The order of enzymes reaction in MT biosynthesis alters the subcellular sites of intermediates and MT formation (Back et al., 2016). For instance, the first and second enzymatic reactions result in the formation of serotonin in the cell endoplasmic reticulum (ER), while the third and fourth enzymatic reaction leads to the formation of serotonin in the cell cytoplasm (Back et al., 2016). The synthesis of MT in the chloroplast occurs when the final step enzyme is SNAT whereas ASMT/COMT is involved in the terminal reaction that occurs in the cytoplasm. Nonetheless, depending on the sites of biosynthesis, both MT and serotonin levels are strongly affected by the capability of anabolic and catabolic flow (Back et al., 2016). TTP and serotonin are significantly accumulated in senesced leaves, while tryptamine and *N*-acetylserotonin are not significantly increased (Back et al., 2016). Thus, these events can be explained by the quick conversion of tryptamine to serotonin by T5H and serotonin conversion *N*-acetylserotonin by SNAT (Kang et al., 2009a,^2010^).

Moreover, a significant accumulation of serotonin is not attained when enzymes competing for serotonin as a substrate are present at the same sub-cellular site. For instance, serotonin is quickly metabolised into phenylpropanoid amides (feruloyl serotonin) by serotonin *N*-hydroxycinnamoyl transferase, which is expressed in the cell cytoplasm (Kang et al., 2009b). Moreover, MT can also be quickly metabolised into 2-hydroxymelatonin (2-OHMel) and cyclic 3-hydroxymelatonin (3-OHMel) by MT-2-hydroxylase (M2H) and melatonin 3-hydroxylase (M3H), respectively, when MT is present in plant chloroplast and cytoplasm, respectively (Byeon and Back, 2015; Lee et al., 2016). In-plant chloroplast MT provides a significant defence to plants against oxidative stresses. The plant chloroplast and mitochondria are significant sites of MT biosynthesis, and it does not preclude the possibility that some MT is not also formed in cell cytosol (Tan and Reiter, 2019). The diverse pathways, along with different sub-cellular sites for MT production, play an important role in the steady-state level of MT and in the induction of MT synthesis in responses to various stresses to cope with adverse impacts (Back et al., 2016).

## Melatonin: The Stress Protectant

**FIGURE 2:**
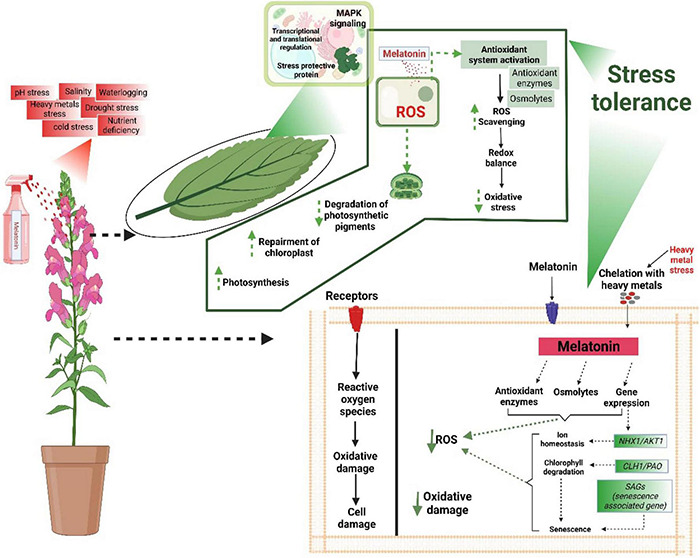
MT being an amphiphilic molecule free crosses cellular membranes and directly scavenges the ROS by increasing the anti-oxidant activities. MT also improves osmolytes accumulation, protects photosynthetic apparatus, maintains redox balance, and affects the signalling transduction and genes expression linked with different stresses to induce stress tolerance.

Melatonin is an excellent antioxidant molecule with the appreciable potential to scavenge ROS and improve stress tolerance (Figure 2). Its exogenous application improves various physiological and biochemical processes and plants’ responses to diverse abiotic stress conditions. It improves chlorophyll contents, photosynthetic efficiency, protein accumulations, and RuBisCO activities and triggers the antioxidant defence system, inducing stress tolerance (Figure 2). MT also stimulates different signalling pathways in response to stress conditions. Here we briefly described the prominent roles of MT mediated tolerance in plants against various abiotic stresses.

## Melatonin Induces Salinity Tolerance in Plants

Salt stress significantly limits crop growth and development and threatens global food production. It mainly induces osmotic stress, ionic and nutritional imbalance, and ROS, resulting in a significant loss in plant growth (Abbasi et al., 2016; Dustgeer et al., 2021; Sultan et al., 2021; Seleiman et al., 2022). Globally, many plant growth regulators (PGR) reported improving salt tolerance to achieve agricultural sustainability (Bastam et al., 2013). Salt stress-induced a reduction in crop productivity by decreasing the photosynthetic efficiency (Meloni et al., 2003). Reduced photosynthetic efficiency can be caused by the closing of stomata and the negative effect of salinity on photosynthetic parameters (Meloni et al., 2003). However, MT application considerably improved the effectiveness of PS-II (Table 1) for photochemical and non-photochemical quenching, which favours increased photosynthetic efficacy under salt stress (Li et al., 2017).

**TABLE 1:** Role of melatonin in inducing salt tolerance in different plant species.

Crops	Salinity stress	MT application	Effects	References
Cotton	150 mM	20 μM	MT supplementation enhanced germination, hypocotyl length, endogenous MT, and regulated the ABA and GA synthesis by mediating the expression of these hormonal-related genes	Chen et al., 2021
Soybean	100 mM	0.10 mM	MT supply increased the chlorophyll synthesis and PS-II activity, upregulated the anti-oxidant defence system and glyoxalase functioning, and reduced MDA accumulation, electrolyte leakage, and lipoxygenase activity	Alharbi et al., 2021
Sugar beet	600 mM	100 μM	MT application improved the seedling growth, root yield, sugar contents, chlorophyll contents, the efficiency of PS-II, and increased the H^+^-pump activities, Na^+^ efflux, K^+^ influx, anti-oxidant activities, and reduced H_2_O_2_ accumulation	Zhang et al., 2021a
Cucumber	150 mM	300 μM	MT application improved photosynthetic efficiency, reduced accumulation of MDA and ROS, and increased the expression of antioxidant genes	Zhang et al., 2020a
Rice	150 mM	200 μM	MT pre-treatment enhanced the seedling biomass production K^+^/Na^+^ ratio, reduced the electrolyte leakage, and increased the activity of nitric oxide synthase (NOS). Moreover, MT also increased the polyamine contents, endogenous MT contents, H^+^-pumps, K^+^ influx, and Na^+^ efflux activities	Yan et al., 2020
Tomato	150 mM	150 μM	The exogenous MT reduced the ROS production maintained the functioning of PS-II, and increased the scavenging of ROS by stimulating antioxidant enzymes	Yin et al., 2019
Oat	150 mM	100 μM	MT application reduced the H_2_O_2_ and MDA accumulation and increased the chlorophyll contents, leaf area, APX, CAT, POS, and SOD upregulated the gene expression	Gao et al., 2019
Wheat	100 mM	1 μM	MT supplementation improved biomass production, IAA content, photosynthetic efficiency, chlorophyll contents, endogenous MT and polyamine contents, and decreased the H_2_O_2_	Ke et al., 2018

MT application regulates the ROS, protecting the photosynthetic apparatus and improving the photosynthetic efficiency and subsequent growth under salt stress, as shown in maize (Chen et al., 2018). MT supply improves sugar accumulation, chlorophyll biosynthesis, and protection of PS-II under salt-stressed conditions (Zhang et al., 2021a). MT supplementation enhances gene expression of various antioxidant, photosynthesis and ROS scavenging enzymes, confining salt tolerance in *Phaseolus vulgaris* and rice (Yan et al., 2021).

Moreover, MT also substantially maintains the ionic balances to counter the salt stress. For example, MT application increased the K^+^ accumulation, decreased the Na^+^ accretion, and kept the higher K^+^/Na^+^ ratio to induce salinity tolerance in maize seedlings (Jiang et al., 2016). The improved ionic homoeostasis in plants is linked with the upregulation of the transcription of the different genes such as *MdNHX1* and *MdAKT1*, which substantially confer the salt tolerance in MT treated *Malus hupehensis* seedlings (Li et al., 2012). Likewise, MT treatment also increased the expression of *NHX1* and *SOS2* in rapeseed seedlings which were associated with a lower Na^+^/K^+^ ratio (Zhao et al., 2018). Moreover, the interaction of Ca^2+^/CaM (Ca^2+^/Calmodulin) and MT is also considered to be involved in overcoming the harmful effects of salt stress. Ca and MT interaction induces long-distance signalling, bringing salt stress tolerance in *Dracocephalum kotschyi* (Vafadar et al., 2020).

Additionally, MT supplementation also caused a reduction in ROS production (Table 1; Wang et al., 2016a; Zheng et al., 2017) through enhanced activities of antioxidant enzymes (APX, CAT, GR, GPX, POD, and SOD) under salt stress (Jiang et al., 2016; Chen et al., 2018). It also increased the actions of the H^+^-pump, which subsequently promoted the K^+^ influx and Na^+^ efflux. It enhanced the activity of antioxidants (APX, CAT, POD, SOD, AsA, and GSH) and the accumulation of soluble sugars, proline, and glycine betaine, favouring the increase in salt tolerance (Zhang et al., 2021a). In conclusion, MT improves plant growth under salt stress by enhancing photosynthetic efficiency, K^+^ influx, and Na^+^ efflux, reducing ROS production, improving antioxidant activities, and accumulating compatible solutes. Therefore, exogenous application of MT can improve salt stress in crops.

## Melatonin Induces Drought Tolerance in Plants

Drought is another significant abiotic stress that considerably limits crop growth and global food production (Hassan et al., 2017, 2020; Mehmood et al., 2021). The reduced water availability induces severe alterations in plant physiological processes, which consequently cause severe yield losses (Hassan et al., 2019, 2021). Melatonin is a potential PGR that confers tolerance in plants against different stress conditions, including drought stress (Meng et al., 2014; Kabiri et al., 2018). Melatonin regulates various physiological, biochemical, and molecular processes (Table 2), which improves the plant’s resistance to stand drought conditions (Campos et al., 2019). The regulation of photosynthetic processes and antioxidant defence system are the main processes controlled by MT under drought stress (Liang et al., 2019). Melatonin protects the photosynthetic apparatus from the effects of drought, which, therefore, improves the photosynthetic efficiency (Meng et al., 2014; Liang et al., 2018).

**TABLE 2:** Role of melatonin in inducing drought stress tolerance in different plant species.

Crops	Stress conditions	MT application	Effects	References
Soybean	30% field capacity	100 μM	MT application improved the photosynthesis and reduced the ABA, MDA, and H_2_O_2_ accumulation by triggering the activities of APX, CAT, POD, and SOD	Imran et al., 2021b
Coffee	40% field capacity	100 μM	MT reduced the chlorophyll degradation, MDA accumulation, electrolyte leakage by increasing the activities of CAT and SOD. Moreover, MT suppressed the expression of chlorophyll degradation gene PAO and upregulated the gene *AREB* encoding ABA-responsive element-binding protein	Cherono et al., 2021
Maize	40% field capacity	100 μM	MT application increased biomass production by reducing the ROS production and increasing the photosynthetic activity and activities of APX, CAT, and POD and accumulation of soluble proteins and proline	Ahmad et al., 2019
*Moringa oleifera*	Drought stress was imposed by skipping irrigation at 45 and 60 days after sowing	150 μM	Foliar application of MT improved moringa’s growth, yield, and quality by enhancing the photosynthetic pigments, phenolic contents, IAA accumulation and reducing the MDA and ROS accumulation by increasing the APX, CAT, and SOD activities	Sadak et al., 2020
Flax	50% field capacity	7.5 mM	MT application improved the growth, yield, photosynthetic activities, IAA contents, soluble sugars, free amino acids, and activities of CAT and POD	Sadak et al., 2020
Wheat	40% field capacity	500 μM	MT improved the photosynthetic rate, efficiency of PS-II, water holding capacity, and activities of APX, DHAR, GPX, GST, and genes expression of these antioxidant enzymes	Zhang et al., 2017a
Alfalfa	Drought stress was imposed by withholding irrigation for seven 7 days	10 μM	MT application reduced the MDA contents ROS production and increased the activities of APX, CAT, GR, and SOD and genes expression	Antoniou et al., 2017
Maize	Drought stress was imposed by withholding irrigation for 7 days	100 μM	MT application improved the photosynthetic activities, stomatal conductance, turgor potential and reduced the MDA and H_2_O_2_ by increasing anti-oxidant activities	Ye et al., 2016

Melatonin also prevents chlorophyll degradation during drought and improves stomatal conductance and photosynthetic efficiency (Liang et al., 2018; Karaca and Cekic, 2019). Moreover, enhanced photosynthetic rate by MT supplementation is attributed to improved PS-II efficiency and better electron transport rates (Zhang et al., 2013; Liang et al., 2018). MT application also protects the chloroplast structure from oxidative stress damage resulting in a substantial increase in photosynthesis (Cui et al., 2017). MT supply also suppressed the expression of chlorophyll degradation genes [pheophorbide a oxygenase (PAO)], which improves the chlorophyll synthesis under stress conditions. MT also increases the expression of photosynthetic genes (RBCS2), thereby improving overall photosynthetic efficiency, assimilating production and crop growth under drought stress (Cherono et al., 2021).

Melatonin application as pre-treatments significantly improved seed germination, delayed senescence, and enhanced root growth under drought stress, resulting in improved plant development and final production (Wang et al., 2013; Zhang et al., 2013). Moreover, MT application also reduced the drought-induced impacts on growth by improving stomatal conductance, photosynthetic efficiency, leaf water status, reducing the electrolyte leakage and H_2_O_2_ accumulation, and increasing soluble sugars and proline accumulation (Liang et al., 2018; Ahmad et al., 2021). The MT mediated protection of the plants from damaging impacts of drought-induced oxidative stress is linked with increased ROS scavenging. The triggered ROS scavenging by MT is due to the stimulated antioxidant defence system under drought stress (Liu et al., 2015b; Cai et al., 2017; Gao et al., 2018; Campos et al., 2019). Water scarcity induced a significant increase in ABA accumulation in plants. Increased ABA level in plants increased oxidative stress linked with lipid peroxidation, electrolyte leakage, and chlorophyll degradation (Jiang et al., 2020). MT supplementation reduced the ABA accumulation under drought stress by downregulating the genes linked with ABA biosynthesis and upregulating the genes involved in ABA catabolism (Jiang et al., 2020). Additionally, in drought-stressed plants, MT appreciably increased the activities of antioxidants (APX, CAT, DHAR, GPX, GR, MDHAR, POD, and SOD) which declined the ROS production safeguarded the plants from drought-induced oxidative stress (Galano et al., 2013; Li et al., 2015; Kabiri et al., 2018; Campos et al., 2019). To summarise, MT improves photosynthetic efficiency, reduces drought-induced ROS production, ABA accumulation, and increases antioxidant activities and proline accumulation, which confer drought tolerance and can be used as a stress protectant under drought stress.

## Melatonin Induces Cold Tolerance in Plants

Cold stress also has devastating impacts on plants and considerably limits crop growth and production (Mishra et al., 2011). Cold stress induces substantial changes in plants’ physiological, molecular, and metabolic activity, altering the membrane permeability and antioxidant activity (Bajwa et al., 2014; Hu et al., 2016). Therefore, MT application improved the cold tolerance of *Bermuda grass* by increasing ROS scavenging (Table 3) through increased antioxidant activities (Shi et al., 2015a). Similarly, spraying the rice seedlings with different MT concentrations (0, 20, or 100 μM) significantly improved the rice growth by preventing ROS MDA accumulation and increasing the efficiency of PS-II (Liu et al., 2015a). The application of MT at lower concentrations (10 and 30 μm) appreciably improved root growth, shoot growth, and biomass production (Bajwa et al., 2014). The application of MT upregulated the cold-responsive genes (COR15a) and antioxidant genes (ZAT10 and ZAT12), which increases the cold tolerance (Bajwa et al., 2014). MT supply also reduced the cold-induced reduction in photosynthetic efficiency by increasing the antioxidant potential and redox homoeostasis, as shown in pea plants (Li et al., 2018). Foliar spray of MT (200 μM) helps in the alleviation of cold-induced growth suppression by improving stomatal conductance, photosynthetic efficiency, the quantum yield of PS-II, and reducing MDA accumulation by increasing CAT, POD, and SOD activities and increasing the expression of antioxidant genes including CmSOD, CmPOD, and CmCAT (Zhang et al., 2017d). The maize seedlings treated with MT (1 mM) under cold stress effectively mitigated the cold stress as shown by enhanced RWC, chlorophyll contents, activities of antioxidants, and lower MDA and H_2_O_2_ accumulation (Turk and Erdal, 2015). Moreover, MT application also induced a significant increase in uptake of nutrients like boron, calcium, copper, iron, potassium, phosphorus, sulphur, and zinc, which generated a considerable increase in maize growth under cold stress (Turk and Erdal, 2015). In conclusion, MT improved cold tolerance by improving photosynthetic activities, stomatal conductance, nutrient uptake, and reduced MDA and H_2_O_2_ through enhanced antioxidant activities and expression of antioxidant genes and has the potential to be used as a stress protectant under cold stress onset.

**TABLE 3:** Role of melatonin in inducing cold stress tolerance in different plant species.

Crops	Stress conditions	MT application	Effects	References
Pistachio	−4°C	0.5 μM	MT supplementation reduced the H2O2 and MDA accumulation, electrolyte leakage, chlorophyll degradation, and activities of APX and GSH	Barand et al., 2020
Tea	−5°C	100 μM	MT foliar spray improved the photosynthetic rate of chlorophyll contents and reduced the ROS accumulation by increasing the anti-oxidant activities and redox homeostasis	Li et al., 2018
Tomato	Day/night temperature of 15/6°C	100 μM	The application of MT reduced the damage to photosynthetic apparatus, increased electron transport, the efficiency of PS-I and PS-II, and protected the membranes from the cold-induced oxidative harms	Yang et al., 2018
Rice	12°C	100 μM	MT alleviated the ROS and MDA accumulation and increased the photosynthetic activity, the efficiency of PS-II, and increased the actions of both enzymatic and non-enzymatic anti-oxidants	Han et al., 2017
Tomato	4°C	100 μM	MT reduced the MDA contents, EL, and increased the activities of antioxidant enzymes and cold-responsive genes	Ding et al., 2017
Barley	4/2°C day/night temperature	10 mM	MT application increased the endogenous MT and increased the photosynthetic efficiency, electron transport, and activities of anti-oxidants	Li et al., 2016b
*Bermuda grass*	4°C	100 μM	MT treatment enhanced the photosynthetic fluorescence parameters and increased carbohydrates and amino acids’ accumulation	Hu et al., 2016
Wheat	Day/night temperature of 5/2°C	1 mM	MT application increased the photosynthetic activities, RuBisCO expression, accumulation of soluble proteins, carbohydrates, and proline and reduced the MDA and ROS accumulation	Turk et al., 2014

## Melatonin Induces Heat Tolerance in Plants

Heat stress (HS) severely restricts plant growth, causes a severe reduction in crop yield, and is considered the most potent food security in this century (Hassan et al., 2021). Therefore, the use of plant growth regulators to protect plants against the adverse effects of this stress is imminent. MT application alleviated the negative impacts of HS (Table 4) and caused a significant increase in growth under HS in various crops (Table 5). MT supplementation maintains the photosynthesis under HS and favours a significant increase in growth (Ahammed et al., 2018). In kiwifruit, it was noticed that MT application effectively modulated the carbon fixation and improved the photosynthesis under HS by genes transcription (Liang et al., 2019). MT-treated seedlings showed increased tolerance to HS due to modulation of antioxidant activities, osmoregulatory system and methylglyoxal detoxification (Li et al., 2019).

**TABLE 4:** Role of melatonin in inducing heat stress tolerance in different plant species.

Crops	Heat stress	MT application	Effects	References
Wheat	40°C	100 μM	MT application reduced oxidative damages by lowering the TBARS and H_2_O_2_ contents and photosynthetic efficacy through enhanced activities of anti-oxidants	Iqbal et al., 2021
Tomato	42°C	10 μM	Exogenous MT increased the chlorophyll fluorescence, electron transport, efficacy of PS-1 and PS-II	Jahan et al., 2021
Wheat	42°C	100 μM	MT reduced the MDA and H_2_O_2_ accumulation and increased proline contents, and activities of APX, CAT, POD, SOD, and GSH and expression of stress-responsive genes (TaMYB80, TaWRKY26, and TaWRKY39)	Buttar et al., 2020
Tomato	42°C	100 μM	MT reduced the heat-induced oxidative stress, lowered the MDA contents, and enhanced the anti-oxidants spermidine and spermine contents and activities	Jahan et al., 2019
Rice	40.6°C	200 μM	MT alleviated the heat-induced damages to photosynthesis chlorophyll and improved the photosynthetic rate by enhancing the anti-oxidant activities	Barman et al., 2019
Kiwifruit	45°C	200 μM	MT pre-treatment ameliorates the head-induced damages by reducing the H_2_O_2_ contents and increasing the proline accumulation, activities, AsA, CAT, POD, SOD, DHAR, and MDHAR, and expression of glutathione S-transferase (GST) genes	Liang et al., 2018
Ryegrass	38/33°C (day/night)	10 μM	MT supplementation reduced the HS-induced leaf senescence. It increased plant height, biomass production, chlorophyll contents, photosynthetic rates, maintained the membrane stability, increased the CK contents, and decreased the ABA contents	Zhang et al., 2017a
Tomato	40°C	10 μM	MT supplementation increased the endogenous MT contents, expression of HSPs, chlorophyll contents and reduced the electrolyte leakage	Xu et al., 2016

**TABLE 5:** Role of melatonin in inducing heavy metals stress tolerance in different plant species.

Crops	Stress conditions	MT application	Effects	References
Spinach	Cd and arsenic stress of 150 mg/kg	100 μM	The application of MT alleviated the Cd and As toxicity, increased the biomass production chlorophyll contents, and reduced lipid peroxidation by increasing the activities of CAT, POD, and SOD activities	Asif et al., 2020
Wheat	Chromium stress 100 mg/kg	2 mM	MT application improved the growth, biomass production, leaf water status, decreased the electrolyte leakage, MDA, and H_2_O_2_ accumulation, and reduced the Cr uptake and accumulation	Seleiman et al., 2020
Tomato	50 μM Nickel stress	100 μM	MT application improved growth, photosynthetic efficiency, chlorophyll contents, decreased the H_2_O_2_ contents Ni accumulation, and upregulated the gene expression of different antioxidants (*SOD*, *CAT*, *APX*, *GR*, *GST*, *MDHAR*, and *DHAR*)	Jahan et al., 2020
Cucumber	30 μM lead stress	150 μM	MT supplementation increased the leaf area, chlorophyll contents, photosynthetic rates, stomatal conductance, transpiration rate, the efficiency of PS-II under Cd stress	Wu et al., 2019
Wheat	200 mM Cd stress	50 mM	MT significantly improved the growth, reduced the MDA and H_2_O_2_ contents, and increased the activities of APX, CAT, GSH, POD, and SOD	Ni et al., 2018
Watermelon	50 mg/L vanadium stress		The application of MT increased the chlorophyll contents, photosynthetic activities, CAT and SOD activities and reduced the MDA and H_2_O_2_ accumulation by regulating the MT biosynthesis genes expression for APX, POD, and SOD	Nawaz et al., 2018
Tobacco	15 μM lead stress	200 μM	MT pre-treatment protected the DNA from lead-induced oxidative damage, increased antioxidant activities, and reduced cell death	Kobyliñska et al., 2017
Tomato	100 mM Cd stress	500 μM	MT increased the H^+^-ATPase activity; antioxidant activities and reduced the Cd accumulation leaves	Hasan et al., 2015

Similarly, wheat MT supplementation suppressed the HS-induced damage by activating antioxidant machinery (Buttar et al., 2020). The supplementation of MT increases SOD activities APX, which counter the ROS and ensure the plants’ survival under HS conditions (Zhang et al., 2017b). Melatonin significantly attenuated HS-induced leaf senescence as indicated by reduced leaf yellowing and increased Fv/Fm ratio, reducing ROS production (Jahan et al., 2021). MT foliar spray also increased the plant growth regulators; for example, endogenous MT and GA contents in heat-stressed plants improved, significantly increasing HS tolerance (Jahan et al., 2021). MT application also reduced the ABA biosynthesis and gene expression, preventing the plants from ABA-induced oxidative damage (Jahan et al., 2021). A recent study indicated that MT supplementation in *Lolium perenne* induced substantial growth by reducing the ABA contents and increasing the endogenous MT and cytokinin contents (Zhang et al., 2017b). The MT application can reduce HS in tomato-induced protein misfolding, thus protecting the proteins from denaturation under HS (Xu et al., 2016). MT also increased the expression of heat shock proteins (HSPs) under HS conditions (Wang et al., 2015; Xu et al., 2016).

Calcium ions play an imperative role against HS tolerance in plants. MT application modulates Ca^2+^ influx through a non-selective Ca^2+^ permeable cation channel (Çelik and Naziroǧlu, 2012), stimulates Ca^2+^ transport across the cellular membranes, and ensures HS tolerance (Santofimia-Castaño et al., 2014). MT also increased the biosynthesis of total phenols and flavonoids, which conferred the HS tolerance (Meng et al., 2018). Therefore, in the light of the findings mentioned above, it is concluded that MT induced the HS by improving the photosynthetic efficiency, protecting the photosynthetic apparatus, reducing ROS and ABA accumulation, and increasing the Ca^2+^ influx antioxidant activities and expression of HSPs. It has enormous potential as a stress protectant used in the exogenous spray.

## Melatonin Induces Ultraviolet Radiation Tolerance in Plants

Ultraviolet (UV) radiations are a severe threat to crop production, and their intensity is continuously increasing due to rapid ozone layer depletion. MT possesses an excellent potential to alleviate UV’s adverse impacts. It has been reported that MT application appreciably facilitated the UV-induced damages to DNA and UV radiations induced ROS in *Nicotiana sylvestris* and *Malus hupehensis* (Zhang et al., 2012; Ullah et al., 2019; Wei et al., 2019; Nazir et al., 2020). MT acts as a potent antioxidant to improve the UV resistance and regulates the expression of different UV signalling pathways, including the ubiquitin-degrading enzyme (COP1), transcription factors (HY5, HYH), and RUP1/2 (Yao et al., 2021). MT supply enhanced the expression of COP1, HY5, HYH, and RUP1/2 which play a significant role in UV-B signalling. Therefore, it regulates the plant antioxidant defence systems to protect them from the damaging impacts of UV-B stress (Yao et al., 2021).

In response to UV stress, endogenous MT accumulation in plant species (Alpine and Mediterranean species) provides UV tolerance (Simopoulos et al., 2005). Likewise, the roots of *Glycyrrhiza uralensis* exposed to UV-B showed a substantial increase in endogenous MT, reducing UV-induced damage to DNA (Zhang et al., 2012). MT application under UV radiation stress increased the endogenous MT and different phenolic compounds, including chlorogenic acid, phloridzin, and quercetin 3-galactoside, which confer UV tolerance (Wei et al., 2019). Though limited studies are conducted to determine the impact of MT against UV stress, more studies are direly needed to underpin the role of MT in mitigating the UV radiation stress in plants.

## Melatonin Induces Waterlogging Tolerance in Plants

Waterlogging has been considered to affect crops’ survival, growth, and production in areas subjected to poor drainage and high rainfalls (Jackson and Colmer, 2005). Waterlogging affects plant growth and development, primarily creating anaerobic conditions and inducing ROS production. MT regulates plant growth and development under different stresses as an excellent antioxidant (Sun et al., 2021). For example, exogenous MT supplementation improved antioxidants activities and reduced the accumulation of MDA and H_2_O_2_ in tomato, pear, and alfalfa for water-logging tolerance (Zhang et al., 2019). Six alfalfa weeds grown under waterlogged conditions of 100 mM MT showed significant improvement in growth, physiological characteristics, photosynthetic efficiency, chlorophyll content, leaf polyamine content, and reduction in MDA and ROS accumulation due to increased antioxidant activity (Zhang et al., 2019). MT also maintains aerobic respiration protects the photosynthetic apparatus from oxidative damage and increases the expression of genes (MbT5H1, MbAANAT3, and MbASMT9) that subsequently improve tolerance to waterlogging stress (Zheng et al., 2017; Gu et al., 2021); for example, treated peach seedlings with MT (200 μM) and found improved chlorophyll concentration, stomatal movements, and reduced electrolyte leakage, lipid peroxidation, and MDA accumulation through increased POD and SOD activity under water deficit stress. MT supplementation enhanced the ADH activity and reserved the transition from aerobic to anaerobic respiration caused by waterlogging (Zheng et al., 2017).

Moreover, MT also controlled the anaerobic respiration enhancing the aerenchyma and suppressing the regulation of metabolic enzymes (Gu et al., 2021). MT improved the tolerance against waterlogging by reducing chlorosis and wilting (Zhang et al., 2013). Another study noted that foliar spray of (100 μ mol L^–1^) substantially enhances the efficiency of PS-II, photosynthetic rate and decreases the MDA accumulation through enhanced antioxidant activities in sorghum (Zhang et al., 2021b). The addition of MT improved waterlogging resistance by increasing the photosynthetic efficiency of photosynthetic pigments and reducing the accumulation of MDA and H_2_O_2_ through increased antioxidant activity. Thus it can be used as a stress protectant against waterlogging stress.

## Melatonin Induces Heavy Metals Stress Tolerance in Plants

Heavy metals (HMs) are a severe threat to global food production. Their concentration in agricultural soil is rapidly increasing due to anthropogenic activities (Hassan et al., 2019; Chattha et al., 2021; Imran et al., 2021a; Rehman et al., 2022). The role of MT to regulate plants grown under different HMs is well explored (Hasan et al., 2015); nonetheless, MT-mediated growth regulation largely depends on MT application rate, heavy metal concentration, and plant species (Table 5). For instance, soybean grown under Al-stress (50 μM) showed a significant increase in growth and antioxidant activities with 1 μM MT compared to the 100 and 200 μM MT (Zhang et al., 2017b). Similarly, red cabbage plants grown under Cu stress showed a significant improvement in growth with 10 μM MT supplementation compared to 100 μM (Posmyk et al., 2008). Conversely, tomato plants grown under Cd stress (100 μM) showed a significant increase in plant growth with MT application of 100 μM as compared to lower rates (Hasan et al., 2015). MT application also reverses the lead-induced cell death and morphological deformation and membrane leakage in stressed plants compared to control (Li et al., 2016a; Kobyliñska et al., 2017).

Melatonin restricts the HM translocation and increases genes expression of MT, thus increasing the endogenous concentration to combat the HM stress (Hasan et al., 2015). Moreover, MT directly scavenges the ROS by improving the antioxidant activities, conferring stress tolerance (Moustafa-Farag et al., 2020). For instance, MT spray enhanced the tolerance against ZnO by increasing the ATPase, RuBisCO, and antioxidant activities in wheat (Zuo et al., 2017). Similarly, MT enhanced the plant tolerance to HMs by modulating the antioxidant enzyme activities (Zhang et al., 2017b). Interestingly, HM induced the upregulation of MT biosynthetic enzymes genes from E. pisciphila tryptophan decarboxylase (EpTDC1 and EpSNAT1) and enhanced the MT biosynthesis improving the tolerance against the HMs in E. coli and A. thaliana (Yu et al., 2021). Strawberry seedlings grown under Cd showed a significant reduction in growth, biomass production, chlorophyll contents and activities of antioxidant enzymes. However, MT application (200 μmol) showed a substantial increase in growth biomass production through enhanced actions of APX, CAT, POD, and SOD, and reduced MDA accumulation (Wu et al., 2021a). MT application also improved the expression of MtPT4 and AM colonisation in Medicago truncatula plants, which improved the overall antioxidant activities and resultantly increased the growth under HM stress (Zhang et al., 2020b). In conclusion, MT alleviated the HMs induced deleterious effects by improving the photosynthetic activity, antioxidant activities, reduced HM uptake and MDA and ROS accumulation. It can be used as a potential stress protectant for managing HMs stress.

## Melatonin Induce Elevated Ozone Tolerance in Plants

Ozone (O_3_) is a highly oxidising pollutant, and increasing O_3_ concentration severely affects plant growth as well as development (Serengil et al., 2011). MT plays an imperative role in plats responses to diverse abiotic stresses; nonetheless, its mechanism in alleviating the O_3_ is poorly understood. Mt crosstalk with various plant growth regulators helps stress alleviation; for example, grape leaves grown under O_3_ were treated with MT modulated ethylene biosynthesis and signalling. O_3_ induced a significant increase in genes expression linked with ethylene biosynthesis, while MT supplementation significantly inhibited the ethylene genes expression (Liu et al., 2021). Further MT application also improved the photosynthetic performance and antioxidant activities under O_3_. The over-expression of MT synthesis gene *VvASMT1* (acetylserotonin methyltransferase 1) also alleviated the O_3_ stress and reduced the ethylene biosynthesis (Liu et al., 2021). The effect of diverse MT concentrations (0, 0.1, 0.5, 2.5, and 12.5 μM) was studied on apple plants grown under O3 stress. The exposure of apple plants to O_3_ induced a significant increase in MDA accumulation. However, MT application reduced the MDA accumulation by increasing the antioxidant activities (CAT, POD, and SOD). Further, MT also improved the accumulation of soluble proteins and non-enzymatic antioxidant activities and conferred the O3 tolerance (Qiu et al., 2019). Therefore, MT induced the O_3_ tolerance by favouring the antioxidant activities and reducing the MDA and ethylene accumulation. However, a wide range of studies is direly needed to underpin the mechanism linked with MT-induced O_3_ stress in plants.

## Melatonin Induces Nutrient Deficiency Tolerance in Plants

The extensive agriculture practices continuously increase the nutrient deficiency problem, and it is considered to aggravate in the coming time. MT possesses an excellent potential to reduce the effects of nutrient deficiency. For instance, MT supplementation significantly increases the iron (Fe) concentration in roots and shoots and alleviates Fe deficiency (Zhou et al., 2016). In another study, MT supplementation enhanced the tolerance of wheat plants to potassium stress (K). MT upregulated the K transporter 1 (TaHAK1) gene expression, improved K absorption, and, therefore, alleviated K deficiency (Li et al., 2021a,b).

Similarly, MT supply reduced ROS production in sulphur (S) deprived plants and mitigated the S-induced deficiency by protecting the macromolecules and ultra-structures (Hasan et al., 2018). MT also promoted the S uptake and assimilation by regulating the genes expression involved in S metabolism and transportation (Hasan et al., 2018). Another investigation indicated the possible mechanism of MT application mediated improvement in growth and physiological parameters by a reduction in the electrolyte leakage, ROS production, and lipid peroxidation through increasing the activities and transcription of antioxidant enzyme genes and improved accumulation of phenols and flavonoids under Fe stress (Ahammed et al., 2020). Here, MT also increased the leaf Fe contents and increased the transcription levels of FRO2 and IRT1, which improved the Fe uptake under Fe deficient conditions (Ahammed et al., 2020). However, other element availability after MT application needs to be investigated. Therefore, nutrient availability can be improved by applying MT through various mechanisms and could be used as a stress protectant under nutrient deficiency.

## Melatonin Induces Soil pH Stress Tolerance in Plants

Soil pH plays a critical role in plants growth, and any fluctuation in soil pH induces stress conditions for plants. MT could help plants withstand the soil fluctuations; for example, MT application improved the growth and yield of tomatoes under alkaline and acid pH stress (Liu et al., 2015a). Soil pH fluctuations can increase the endogenous MT and are reported to be increased by 12 times under pH stress in untreated plants (Arnao and Hernández-Ruiz, 2013). MT supplementation (0.1 and 1 μM) in soybean mitigated the Al-induced toxicity in acid soils through an enhanced accumulation of osmolytes and antioxidant activities (Zhang et al., 2017b). MT induced pH stress tolerance by activating MT receptors (MTNR1A and MTNR1B) and improving antioxidant defence (Arnao and Hernández-Ruiz, 2006).

Besides this, MT under alkaline stress also increased the accumulation of polyamines which conferred stress tolerance (Gong et al., 2017). MT also reduced oxidative stress, and membrane leakage in alkaline stressed conditions by scavenging the ROS (Hardeland, 2013; Gong et al., 2017). The increase in antioxidant activities preserves chloroplast grana, prevents chlorophyll degradation, and improves photosynthesis under alkaline stress (Debnath et al., 2018). MT’s protective role under sodic alkaline stress is also linked with NO signalling. Under alkaline stress, MT triggers NO accumulation by downregulation of expression of S-nitrosoglutathione reductase (Corpas and Barroso, 2015; Kaur et al., 2015; Wen et al., 2016). These findings suggested that NO is a downstream signal in plants’ tolerance to alkaline stress (Liu et al., 2015b). Similarly, MT application significantly improved the expression of acetyltransferase NSI-like genes and lowered the production of H_2_O_2_ under acidic soils (Moustafa-Farag et al., 2020). Little research is done on the ameliorative effect of exogenous MT in the context of plants grown under pH stress. Nonetheless, more studies are required to explore the mechanistic pathways of MT in inducing pH stress tolerance.

## Mechanism of Melatonin Induced Stress Tolerance

### Melatonin Mediated Upgrading of the Antioxidant Defence System Under Stress Conditions

Plants have different physiological and biochemical adaptations to cope with various abiotic stresses. ROS are produced in plants under other stress conditions (Hassan et al., 2019), which induce oxidative stress and cause damage to macromolecules and biological structures (Imran et al., 2021b; Iqbal et al., 2021). Thus, plants activate antioxidant defence systems to counter the deleterious impacts of abiotic stresses (Iqbal et al., 2021). MT is an excellent molecule that improves plant growth by triggering the antioxidant enzymes under stressed conditions (Table 6).

**TABLE 6:** Effect of MT application on anti-oxidant defence system under different stress conditions.

Plant species	Stress conditions	MT application	Effect on anti-oxidant	References
**Salt stress**				
Cotton	100 mM	200 μM	↑ APX and POD	Zhang et al., 2021c
Cotton	150 mM	200 μM	↑ APX, CAT, POD, and SOD	Jiang et al., 2020
Maize	150 mM	20 μM	↑ APX, GR, GPX, POD, and SOD	Chen et al., 2018
**Drought stress**				
Maize	40% field capacity	150 μM	↑ APX, CAT, POD, and SOD	Ahmad et al., 2021
Rice	Irrigation was withhold	100 μM	↑ APX, GPX, and POD	Silalert and Pattanagul, 2021
Rapeseed	35–40% field capacity		↑ AsA, APX, CAT, GSH, POD, and SOD	Khan et al., 2020b
**Cold stress**				
Tea	4°C	100 μM	↑ APX, AsA, CAT, GSH, POD, and SOD	Li et al., 2019
Rice	12°C	150 μM	↑ CAT, GSH, and SOD	Han et al., 2017
Cucumber	10°C	500 μM	↑ CAT, GR, POD, and SOD	Marta et al., 2016
Heat stress				
Soybean	42°C	100 μM	↑ AsA, CAT, and SOD	Imran et al., 2021b
Wheat	40°C	100 μM	↑ APX, CAT, POD, and SOD	Buttar et al., 2020
Tomato	42°C	100 μM	↑ APX, CAT, POD, GR, and MDHAR	Jahan et al., 2019
**Metal stress**				
Strawberry	Cd 300 mM	200 μM	↑ APX, CAT, POD, and SOD	Wu et al., 2021a
Tea	As 25 μM	100 μM	↑ APX, CAT, POD, and SOD	Li et al., 2021b
*Bermuda grass*	Pb 2000 mg kg^–1^	100 μM	↑ AsA, APX, CAT, GT, POD, and SOD	Xie et al., 2018

Drought and salt stress-induced ROS production was regulated by different plant growth regulators. These ROS act as plants’ internal defence systems to trigger the scavenging of ROS and reduce oxidative stress by increasing the activities of antioxidant enzymes (Liang et al., 2019).

Melatonin is a multi-functional antioxidant, and it substantially scavenges the ROS and improves stress tolerance (Arnao and Hernández-Ruiz, 2014). MT stimulates the enzymatic antioxidative defence system and protects against stress conditions (Ye et al., 2016). It also promotes the ABA degradation enzymes and scavenges the ROS by increasing the activities of APX, CAT, DHAR, GPX, GR, and SOD (Kabiri et al., 2018; Campos et al., 2019; Li et al., 2021a). Under salt stress, MT application significantly increased the photosynthetic rate and reduced oxidative stress, as discussed earlier (Zhang et al., 2017c). The MT-induced protection under salt stress is linked with improved light absorption, electron transport, the efficiency of PS-II, and reduction in oxidative stress induced by increase in activities of antioxidants (AsA, CAT, GSH, POD, and SOD) in melon crop (Zhang et al., 2017c).

Similarly, the application of MT under HS significantly increased proline accumulation. It reduced the MDA and H_2_O_2_ accumulation through the improved activity of CAT, POD, and SOD and the expression of genes linked with these enzymes (Jahan et al., 2019; Buttar et al., 2020). Similarly, MT application substantially improved the working of APX, CAT, POD, SOD, and GSH and, therefore, decreased ROS accumulation under HM and HS stress (Byeon et al., 2015; Hasan et al., 2015). MT also reduces the excessive ROS production induced by HM in rice, wheat, and watermelon by activating the SOD (Lee and Back, 2017; Nawaz et al., 2018). Similarly, other authors also reported that MT significantly improved the activities of APX, CAT, POD, SOD, and other antioxidant activities under waterlogging, cold, and ozone stress (Zhang et al., 2017c; Qiu et al., 2019; Gu et al., 2021). Thus, all these findings endorsed that MT supplementation effectively up-graded the antioxidant defence system to alleviate the effects of different abiotic stresses.

### Interaction and Crosstalk of Melatonin With Other Hormones

Hormones play a critical role in plant growth and MT is widely involved in the metabolism of a range of hormones, including IAA, ABA, gibberellic acid (GA), cytokinin (CK), and ethylene (Arnao and Hernández-Ruiz, 2018). MT has similar chemical properties to IAA, and both these two hormones use tryptophan in their biosynthesis pathways as substrate (Wang et al., 2016b). MT acts as a growth regulator and it shows IAA-like activities (Pelagio-Flores et al., 2012). MT improves root development and vegetative growth in different crops, including *Arabidopsis*, barley, maize, rice, and tomato (Arnao and Hernández-Ruiz, 2018). MT regulates the formation of a root by IAA independent pathway in *Arabidopsis* (Pelagio-Flores et al., 2012).

Conversely, crosstalk between IAA and MT was also reported; for instance, an increase in endogenous IAA was reported in *Brassica* with external application of MT (Chen et al., 2009; Arnao and Hernández-Ruiz, 2018). Further application of IAA significantly improved the endogenous MT (Wang et al., 2016a). MT mediates mediate the biosynthesis of ABA, and it regulates the ABA metabolism, thus reducing the ABA accumulation under stress conditions. For instance, in apples, MT downregulated the *MdNCED3*, an essential ABA biosynthesis gene, consequently decreasing the ABA accumulation (Li et al., 2015). Likewise, MT downregulated the ABA under HS in perennial ryegrass and reduced the ABA contents (Zhang et al., 2017b). Similarly, MT also downregulated ABA signalling and improves stress tolerance (Fu et al., 2017). Interestingly MT also increased the expression of cold-responsive genes and reduced the ABA accumulation, therefore considerably increasing the cold tolerance (Fu et al., 2017).

Exogenous MT also ameliorated the impacts of salinity stress by regulating ABA biosynthesis and catabolism. In salty conditions, MT reduced the transcript levels of ABA synthesis-related genes (*CsNCED1* and *CsNCED2*), which resulted in a reduction in ABA accumulation under stress conditions. Moreover, MT application increased the expression of genes (GA20ox and GA3ox) involved in GA, enhancing the GA accumulation under stress conditions (Zhang et al., 2014). In another study Zhang et al. (2017b) noted that MT induced CK activation and inhibition of ABA biosynthesis significantly inhibited the leaf senescence in ryegrass plants grown under HS. All this evidence suggests that MT can be a potential signalling molecule that triggers signalling transduction and improves plant growth and development under stress conditions.

### Success Stories: Engineered Melatonin Biosynthesis to Enhance Abiotic Stress Tolerance

Melatonin is a natural hormone in plants and protects them against stress conditions. Thus, increasing the endogenous MT is crucial to combat the effects of abiotic stresses (Table 7). The transgenic strategy is an effective strategy to improve the endogenous MT level. Nonetheless, over-expression of MT responsive genes under various abiotic stresses is studied in few crops. Many studies reported that MT levels significantly increased under stress conditions (Xing et al., 2021; Qari et al., 2022).

**TABLE 7:** Role of melatonin in inducing stress tolerance in transgenic plant species.

Crop species	Genes	Stress	Characteristics	References
Tomato	*SlCOMT1*	Salt stress	The over-expression of *SlCOMT1* genes enhanced the crop growth, biomass production, proline contents and reduced the H_2_O_2_ contents by increasing the activities of SOD	Liu et al., 2019
Alfalfa	*MsSNAT*	Cadmium stress	The increase in expression of *MsSNAT* increased the endogenous MT, root length, chlorophyll contents and decreased the H_2_O_2_ accumulation Cd accumulation in plant roots	Gu et al., 2017
Switch grass	*oAANAT; oHIOMT*	Salt stress	The increase in genes expression increased the plant height, stress growth, proline contents, leaf water status, and decreased MDA accumulation, electrolyte leakage, and Na^+^ accumulation	Huang et al., 2017
Tomato	*oHIOMT*	Drought stress	The overexpression of oHIOMT increased the drought tolerance and decreased the leaf wilting and dehydration rate	Wang et al., 2014
Tobacco	*MzASMT 1*	Salt stress	The over-expression of the MzASMT 1 gene increased the MT contents, plant height, biomass production, leaf water status, chlorophyll contents, proline accumulation, and reduced the MDA contents by increasing activities of anti-oxidants	Zhuang et al., 2020
*Arabidopsis*	*TaCOMT*	Drought stress	Over-expression of TaCOMT increased GA and IAA accumulation, decreased ABA accumulation, increased endogenous MT accumulation	Yang et al., 2019
*Arabidopsis*	*VvSNAT1*	Salt tolerance	The over-expression of *VvSNAT1* increased the endogenous MT contents, reduced leaf wilting, increased germination and biomass production, and decreased the MDA and H_2_O_2_ accumulation	Wu et al., 2021b

Enzymes like AANAT and HIOMT are essential for the biosynthesis of MT, and the over-expression of these enzymes in tomatoes under drought stress increased the endogenous MT level (Wang et al., 2014). Higher MT levels improve the plant’s growth and tolerance to change, and resistance to drought and pesticides (Campos et al., 2019; Yan et al., 2019). For instance, in *Arabidopsis*, higher expression of FIT1, FRO2, and IRT1 genes after MT application restored the Fe deficiency (Zhou et al., 2016). In another study, the over-expression ASMT gene increased the endogenous MT level and provided cellular protection by increasing the expression of HSPs and triggering the HS tolerance (Xu et al., 2016). Moreover, in tomato over-expression of the HsfA1a gene, the COMT1 transcription factor was upregulated, which increased the MT biosynthesis and resistance against the Cd stress (Choi et al., 2017). Likewise, in rice crops, overexpression of chloroplast caffeic acid O-methyltransferase (COMT) increased the MT contents and improved the seedling growth under stress conditions (Choi et al., 2017).

Over-expression of MT biosynthesis pathway genes such as tryptophan decarboxylase-interacting protein 2 (*MeTDC2*), N-acetylserotonin O-methyltransferase-interacting protein 2 (*MeASMT2*), and N-acetylserotonin O-methyltransferase 3 (*MeASMT3*) significantly increased endogenous MT and improved stress tolerance (Wei et al., 2018). Ma et al. (2017) used the bacterium *Pseudomonas fluorescens* RG11 strain to increase the endogenous MT in grapes, which increased the salt tolerance in grapes and reduced the cellular damage by decreasing the ROS production (Ma et al., 2017). Moreover, a bacterial strain (*Bacillus amyloliquefaciens*) from grapevine roots significantly increased the endogenous MT production and facilitated the adverse impacts of drought by H_2_O_2_ scavenging (Jiao et al., 2016). Thus, all these findings suggested that a transgenic increase in endogenous MT could be a promising approach to improving stress tolerance.

## Conclusion and Future Perspectives

Melatonin has excellent properties for improving tolerance to abiotic stress. Melatonin alters different biochemical, molecular and physiological processes to induce stress tolerance in plants. MT protected the photosynthetic apparatus from oxidative damage caused by stress and increased the efficiency of photosynthesis. In addition, melatonin also stimulates cell signalling that controls diverse physiological and molecular aspects to confer stress tolerance in plants. Application of MT under various stresses reduced ROS production by activating antioxidant enzymes, accumulating compatible solutes, and increasing the expression of stress-responsive genes. However, many questions need to be answered by conducting a wide range of studies.

Future studies need to study the anatomical changes in leaves and roots of MT plants under different stresses. Similarly, researchers need to investigate the effect of MT application on fruit set, pollen viability, and abscission. The precise role of MT in signalling pathways under different stresses needs to be investigated. Other studies have reported the interaction of MT with different osmolytes and hormones. However, further studies are required to support the exchanges and interactions of MT with other osmolytes and hormones in individuals and combinations of various stresses. In addition, investigating the role of MT under different stresses would also unravel the potential of protecting spray in other crops. Recent improvements in plant genomics, transcriptomic, proteomic, and metabolomic will also to better understand hormone networks and their interaction and crosstalk under different stresses.

Regulation of gene expression and interactions with different hormones is also a crucial factor in MT that significantly increases stress tolerance. However, endogenous MT is not sufficient to cope with challenging conditions. Under such conditions, exogenous MT is resorted to increase endogenous MT to maintain average growth under stressful conditions. However, the cellular signalling pathways induced by MT require more profound studies in different crops under signal and combination of various stresses. ROS are mainly produced in plant chloroplast and mitochondria. Because MT functions as a signalling molecule, it would be interesting to study inter-organelle MT signalling under different stresses. In addition, the molecular mechanism of MT to increase the expression of antioxidant and stress-responsive genes should be investigated in more detail. Engineering MT signalling will open new perspectives on current knowledge to understand MT-induced stress tolerance. The effects of MT under nutrient deficiency, UV irradiation, and ozone stress are not fully explored. Therefore, a deeper understanding of MT under nutrient deficiency, UV, ozone, and pH stress needs further exploration. More intensive transcriptomic and proteomic studies would reveal how MT are affected by nutrient deficiency, UV radiation, ozone, and pH stress. Finally, the patterns of MT application in plant responses to individual and combined stresses under field conditions should also be investigated.

## Author Contributions

MUH and GH conceived the idea. MUH prepared the original draft of manuscript. AM, MIA, RM, MA, HA, GH, MS, MB, SP, AES, and SHQ reviewed and edited the final version. GH supervised the study and provided funding. All authors contributed to the article and approved the submitted version.

## Conflict of Interest

The authors declare that the research was conducted in the absence of any commercial or financial relationships that could be construed as a potential conflict of interest.

## Publisher’s Note

All claims expressed in this article are solely those of the authors and do not necessarily represent those of their affiliated organizations, or those of the publisher, the editors and the reviewers. Any product that may be evaluated in this article, or claim that may be made by its manufacturer, is not guaranteed or endorsed by the publisher.
